# Dietary behaviors and physical fitness among Chinese adolescents aged 13–16 years: a comparative study on breakfast, eggs, dairy, and sugar-sweetened beverages by urban–rural location and sex

**DOI:** 10.3389/fnut.2026.1724764

**Published:** 2026-01-28

**Authors:** Youjia Li, Shaokai He, Liangsen Wang, Wenyue Ma, Wenfei Zhu, Ying Hou, Yuliang Sun

**Affiliations:** 1 School of Physical Education, Shaanxi Normal University, Xi’an, China; 2 Fuzhou Preschool Education College, Fuzhou, China

**Keywords:** adolescents, Chinese, dietary behaviors, physical fitness, urban–rural

## Abstract

**Background:**

This study aims to investigate the associations between four dietary behaviors and physical fitness among Chinese adolescents aged 13–16, with particular attention to urban–rural and sex-related differences.

**Methods:**

The data were obtained from the Chinese National Survey on Students’ Constitution and Health (CNSSCH). The analysis included 43,194 participants aged 13–16 from urban and rural China. Multivariable linear regression analyses were used to examine associations between dietary and physical fitness, adjusting for physical activity, sleep duration, and sedentary time.

**Results:**

More frequent consumption of breakfast, eggs, and dairy products was linked to better physical fitness outcomes in all groups. Higher intake of sugar-sweetened beverages (SSBs) was linked to poorer fitness performance. These relationships were stronger in rural adolescents, especially for strength (*β* = 0.047, *p* < 0.001) and endurance (*β* = −0.063, *p* < 0.001). The associations were more evident among girls.

**Conclusion:**

Dietary behaviors were related to physical fitness in adolescents aged 13–16. The relationships were stronger in rural areas than in urban areas. Regular intake of breakfast, eggs, and dairy products was linked to better strength, speed, and endurance. Overall, higher consumption of breakfast, eggs, and milk, and reduced intake of SSBs were associated with modestly better physical fitness outcomes among adolescents.

## 1 Introduction

Physical fitness is a state of health characterized by the integrated capacity of bodily systems to sustain optimal physiological function and to execute a range of motor tasks during physical exertion (1). Physical fitness is widely seen as an important part of adolescent health (2). Studies show that fitness levels during adolescence are associated with both current and later health outcomes, including obesity risk, bone health, and cardiovascular disease (1, 3). For this reason, physical fitness plays an important role in adolescent growth and long-term health, and diet is considered one of the modifiable factors (4). Physical capacity includes cardiorespiratory endurance, speed, flexibility, muscular strength, and body composition (5). They are commonly used in school fitness tests in China (6, 7). Among them, body composition is more strongly influenced by dietary behaviors during adolescence.

Many studies have shown that dietary behaviors are associated with physical fitness in adolescents (8–11). Regular intake of breakfast, eggs, and dairy products is related to healthier body mass index (BMI) and better muscle strength and endurance (12). However, skipping breakfast was associated with poorer physical fitness, particularly in muscular strength and endurance (13). High intake of sugar-sweetened beverages (SSBs) is linked to lower cardiorespiratory fitness and a higher risk of obesity (14, 15). Studies in Chinese adolescents also report that greater SSB consumption is related to poorer fitness levels and less physical activity, especially when screen time is high (16). These studies indicate associations between adolescents’ dietary behaviors and physical fitness. However, most previous studies have primarily examined the association between healthy eating and specific aspects of physical fitness. In addition, many studies have been limited to urban areas in developed regions (15, 17). Few studies using national data have examined how multiple dietary behaviors relate to overall physical fitness among Chinese adolescents in both urban and rural regions (18).

Dietary habits in China have changed over the past decades. Diets have shifted from a mainly cereal-based pattern to a more varied intake of foods, including more protein, dairy products, fruits, and vegetables. These changes have improved overall nutritional status (19, 20). Dietary habits in China have changed over the past decades. Diets have shifted from a mainly cereal-based pattern to a more varied intake of foods, including more protein, dairy products, fruits, and vegetables. These changes have improved overall nutritional status (21–23). Dietary behaviors still differ across regions in China. Studies show clear differences in food choices and nutritional patterns among children and adolescents (24). Differences between urban and rural areas have increased in recent years (25, 26). These differences are related to location, income, and access to food. Rural populations often maintain more traditional diets, with higher intakes of plant-based foods and lower intakes of animal products than urban populations (19). For this reason, it is important to study dietary behaviors in both urban and rural areas (17).

Therefore, the purpose of this study was to investigate the relationships between diverse physical fitness and the frequency of consumption of breakfast, eggs, dairy products, and SSBs among Chinese teenagers aged 13–16 years. These dietary behaviors were chosen because they are included in the Chinese Dietary Guidelines and are commonly measured in school surveys. The study also compares these relationships between urban and rural adolescents.

## 2 Methods

### 2.1 Study overview and participants

**Figure 1:**
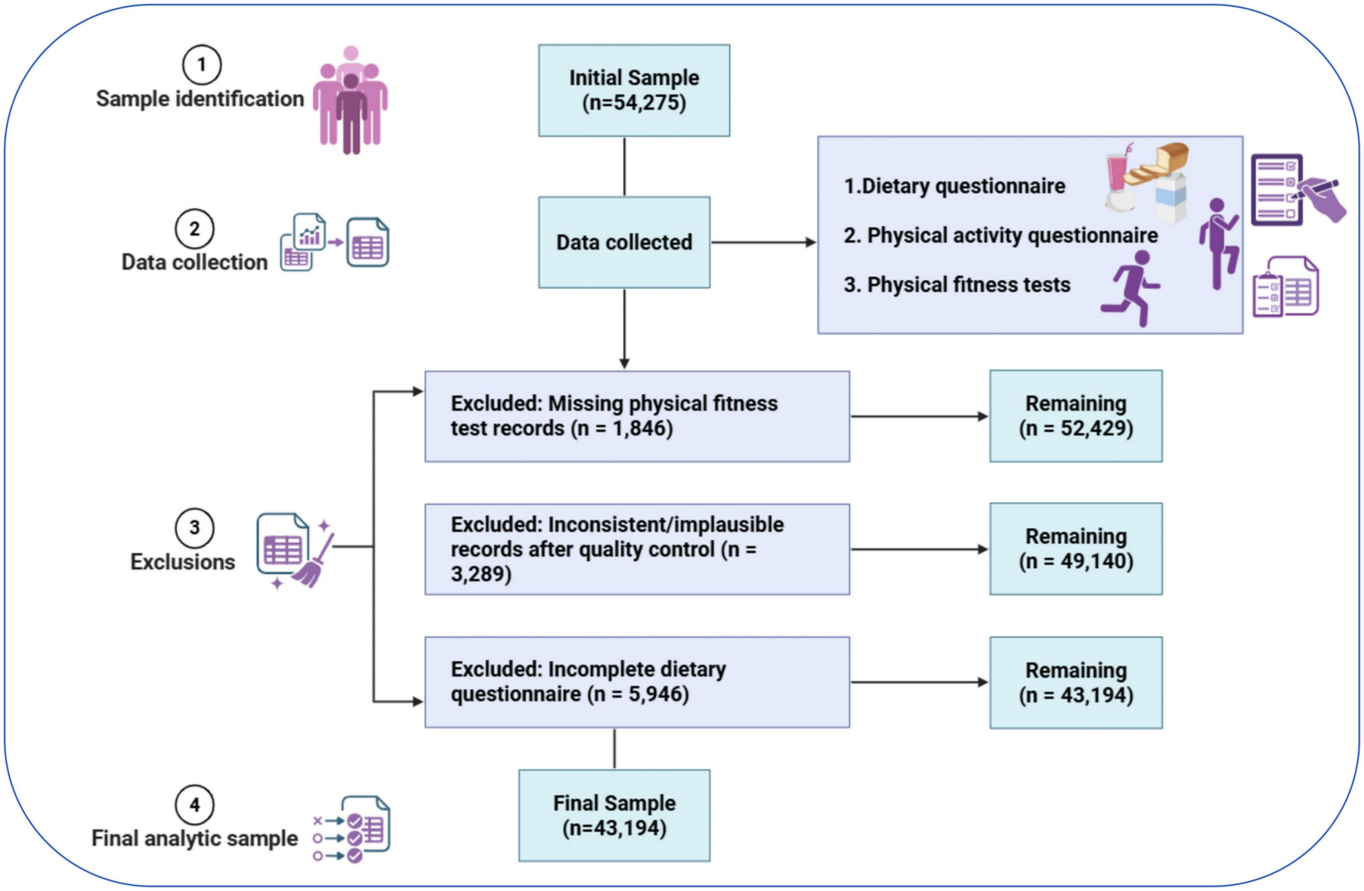
Participant inclusion flowchart and study design schematic.

Data for this analysis were drawn from the 2019 Chinese National Survey on Students’ Constitution and Health (CNSSCH). This nationwide survey collects standardized information on physical fitness and general health among adolescents in China (27). In 2019, a sample of 54,275 adolescents aged 13–16 years was selected using stratified sampling from 31 provinces in China. The sample was divided into urban and rural groups. Physical fitness was measured with standard field tests. Dietary behaviors and physical activity were measured using self-reported questionnaires (3). For our analysis, we focus on a sample of 43,194 participants after excluding those missing specific covariates or incomplete questionnaires. Ethical approval for this study was obtained from the Ethics Committee of Shaanxi Normal University (Approval No.: 201916001, Date: September 2019) (see Figure 1).

### 2.2 Measurement of physical fitness

Physical fitness was evaluated in several areas (28). All tests followed the procedures described in the 2019 National Student Physical Fitness and Health Research Manual (27, 29, 30).

Body morphology was assessed using BMI (kg/m^2^). BMI = weight (kg)/height^2^ (m^2^).

Cardiorespiratory capacity was evaluated through forced vital capacity (FVC, mL) (31). Participants stood upright and held the handle of the spirometer. They took a deep breath and then exhaled steadily until no more air could be released.

Speed was measured using a 50 m sprint (seconds). Participants ran a straight 50 m distance at full effort. The time was recorded using a stopwatch.

Flexibility was evaluated using the sit-and-reach test (cm). Participants kept their knees straight and bent forward. The middle fingers of both hands were used to push the slider forward at a steady speed until it could not move further.

Strength assessments included the standing long jump (cm), chin-ups for boys, and 1-min sit-ups for girls. During the standing long jump test, participants positioned themselves behind the starting line, kept their feet apart, and performed a forward jump with both legs. The distance from the take-off line to the nearest landing point was recorded. For the sit-up test, participants lay on a mat with their hands behind their heads and their legs bent at a 90-degree angle. Another person held the ankles. One sit-up was counted when the elbows reached the knees. The number of sit-ups completed in 1 min was recorded. For the chin-up test, participants held a high bar with hands shoulder-width apart. One repetition was counted when the chin passed above the bar and the body returned to the hanging position. The total number of chin-ups was recorded.

Endurance was evaluated by timed runs (1,000 m for boys and 800 m for girls, in seconds) (7, 32). Participants ran the required distance as fast as possible. The finishing time was used to reflect aerobic endurance. The test followed sex-specific rules defined in the national protocol to match physical differences during adolescence and ensure safe testing (15).

### 2.3 Questionnaire assessment of dietary behaviors and physical activity duration

The dietary questionnaire used in this study was based on the survey used in the 2014 China National Survey on Students’ Constitution and Health (CNSSCH). Previous studies have shown that this questionnaire is reliable and valid (27, 29). The questionnaire was designed for use in different regions of China, including remote and less developed areas. The questions were kept simple and could be used nationwide. This approach also helped improve response rates and reduce possible bias in the data. The specific key questions are as follows:

(1) How many days did you eat breakfast in the last 7 days? (33–35).
(2) How many days did you eat at least one egg in the last 7 days?
(3) How many days in the last 7 days did you consume at least one glass of milk, yogurt, or soy milk?
(4) How many times a day did you typically consume sugar-sweetened beverages, such as cola, tea drinks, fruit juice drinks, etc., during the previous 30 days?

For items 1–3, participants reported how often they consumed each food by choosing the number of days per week, from 0 to 7. Item 4 used seven response categories. These included options such as “none,” “less than once per day,” “once per day,” and up to “five or more times per day” (29).

Physical activity levels, sleep duration, and sedentary time were measured with a questionnaire that has been used before. We also used this same tool in our earlier studies (36). The time spent in moderate-to-vigorous physical activity (MVPA), sleep duration, and sedentary time all came from the participants’ own reports using this questionnaire. Specifically, the questionnaire asked: “Over the past 7 days, on how many days did you do light, moderate, or vigorous physical activities?” and “How long did you usually do these activities each day (in minutes)?” Participants also reported their typical bedtime and wake-up time, which we used to calculate their average daily sleep duration. For sedentary time, they were asked to report how many hours per day they usually spent sitting or doing other inactive things over the past week.

### 2.4 Statistical analysis

The R software (version 4.4.3; R Foundation for Statistical Computing, Vienna, Austria) was used for all statistical analyse (37). Continuous variables were first evaluated for normality using kernel density estimation, and extreme outliers (*z*-scores > |3.0|) were eliminated. Complete-case analysis was used to manage missing data; individuals who had missing values in important variables were not included in the relevant analyses. Participants were classified by household registration status (urban/rural) and sex (boys/girls). Group differences in dietary behaviors and physical fitness outcomes were tested using independent-sample t tests, with statistical significance set at *p* < 0.05. Multivariable linear regression models were used to analyze the associations between four dietary behaviors and physical fitness. The models were adjusted for potential confounders, including physical activity, sleep duration, and sedentary time.

## 3 Results

### 3.1 Participant characteristics

The study included a total of 43,194 adolescents aged 13–16 years, stratified by urban/rural residence and sex: urban boys (*n* = 10,019, 23.2%), rural boys (*n* = 10,102, 23.4%), urban girls (*n* = 11,499, 26.6%), and rural girls (*n* = 11,574, 26.8%) (Table 1).

**Table 1:** Sex distribution by urban–rural residence among adolescents.

Group	Boys	Girls	Total
Urban	10,019	11,499	21,518
Rural	10,102	11,574	21,676
Total	20,121	23,073	43,194

### 3.2 Comparison of four dietary behaviors and physical fitness between urban and rural adolescents

Significant urban -rural disparities were observed in four dietary behaviors and physical fitness (Table 2). Among boy students, urban residents reported significantly higher frequency of consumption of breakfast (*p* < 0.001, *d* = 0.10), eggs (*p* < 0.001, *d* = 0.162), dairy products (*p* < 0.001, *d* = 0.238), and significantly lower sugary beverage consumption (*p* < 0.001, *d* = −0.052). Additionally, they exhibited higher BMI values (*p* < 0.001, *d* = 0.058) than their rural counterparts. Additionally, urban boys exhibited greater FVC than rural boys (*p* < 0.001, *d* = 0.081). However, urban boys performed significantly poorer than rural boys in both the sit-and-reach test (*p* < 0.001, *d* = −0.065) and the chin-up test (*p* < 0.001, *d* = −0.061). Additionally, urban boys engaged in significantly more time in physical activity than rural boys (*p* < 0.001, *d* = 0.084). Urban girl students demonstrated significantly better performance in FVC (*p* < 0.001, d = 0.108) and sit-up tests (*p* < 0.001, *d* = 0.175), along with higher consumption frequencies of breakfast (*p* < 0.001, *d* = 0.109), eggs (*p* < 0.001, *d* = 0.167), and dairy products (*p* < 0.001, *d* = 0.217). However, urban girls had longer 800 m running times than rural girls (*p* < 0.001, *d* = 0.079). Additionally, urban girls exhibited a slightly but statistically higher BMI than their rural counterparts (*p* = 0.046, *d* = 0.026).

**Table 2:** Comparison of four dietary behaviors and physical fitness among urban and rural adolescents aged 13–16.

Variable	Sex	Urban (Mean ± SD)	Rural (Mean ± SD)	*p*	*d*
BMI (kg/m^2^)	Boys	19.69 ± 2.44	19.55 ± 2.37	**<0.001**	0.058
	Girls	20.14 ± 2.38	20.08 ± 2.39	**0.046**	0.026
FVC (mL)	Boys	3329.96 ± 809.22	3265.16 ± 783.31	**<0.001**	0.081
	Girls	2490.37 ± 552.33	2432.01 ± 531.20	**<0.001**	0.108
Sit-and-reach (cm)	Boys	8.95 ± 6.41	9.37 ± 6.44	**<0.001**	−0.065
	Girls	12.80 ± 5.95	12.77 ± 5.92	0.701	0.005
Standing long jump (cm)	Boys	205.98 ± 26.87	206.56 ± 26.81	0.126	−0.022
	Girls	159.76 ± 20.53	160.25 ± 20.99	0.073	−0.024
Chin-ups/1 min sit-ups (reps)	Boys (chin-ups)	3.42 ± 4.07	3.66 ± 4.05	**<0.001**	−0.061
	Girls (sit-ups)	33.57 ± 9.72	31.86 ± 9.83	**<0.001**	0.175
50-m sprint (s)	Boys	7.91 ± 0.70	7.91 ± 0.72	0.968	−0.001
	Girls	9.38 ± 0.81	9.39 ± 0.83	0.309	−0.013
Endurance run (s)	Boys (1,000 m)	262.95 ± 31.55	263.01 ± 32.27	0.897	−0.002
	Girls (800 m)	250.87 ± 27.74	248.66 ± 28.41	**<0.001**	0.079
MVPA (min/week)	Boys	236.40 ± 116.67	226.76 ± 112.91	**<0.001**	0.084
	Girls	209.10 ± 109.89	208.32 ± 109.32	0.589	0.007
Breakfast frequency (days/week)	Boys	6.09 ± 1.81	5.91 ± 1.90	**<0.001**	0.1
	Girls	6.07 ± 1.73	5.88 ± 1.92	**<0.001**	0.109
Egg consumption (days/week)	Boys	3.21 ± 2.42	2.83 ± 2.35	**<0.001**	0.162
	Girls	2.90 ± 2.34	2.52 ± 2.27	**<0.001**	0.167
Soy milk/yogurt (days/week)	Boys	4.38 ± 2.45	3.79 ± 2.48	**<0.001**	0.238
	Girls	4.22 ± 2.36	3.70 ± 2.44	**<0.001**	0.217
Sugary beverage consumption (times/day, past 30 days)	Boys	2.29 ± 1.12	2.35 ± 1.18	**<0.001**	−0.052
	Girls	2.15 ± 0.94	2.14 ± 1.01	0.665	0.006

M, mean; SD, standard deviation; BMI, body mass index; FVC, forced vital capacity; MVPA, moderate-to-vigorous physical activity; breakfast frequency (days/week), number of breakfast days in the past 7 days; egg consumption (days/week), number of days eating eggs in the past 7 days; soy milk/yogurt (days/week), soy milk/yogurt (days/week); sugar-sweetened beverages consumption (times/day, past 30 days), number of sugared beverages per day in the past 30 days. Bold values indicate statistical significance (*p* < 0.05).

### 3.3 Four dietary behaviors and physical fitness in urban versus rural adolescents

After adjusting for MVPA, sleep duration, and sedentary time, modest but consistent links have been shown between the four dietary factors and physical fitness. These results are provided in Supplementary Table S1.

In the urban group, breakfast consumption frequency in boys was independently and positively associated with standing long jump performance. Among urban girls, breakfast intake was positively associated with physical fitness, including the sit-and-reach test and the standing long jump. In contrast, inverse associations were observed for the 800 m running. In the rural group, distinct behaviors were observed between boys and girls. Among rural boys, breakfast consumption frequency was positively associated with standing long jump and sit-and-reach performance, while no significant associations were observed with FVC or running-related indicators. In contrast, rural girls showed a broader behavior of associations, with breakfast consumption positively associated with FVC, standing long jump, sit-and-reach performance, and 1-min sit-ups, and negatively associated with both the 50 m sprint and the 800 m run. However, all standardized regression coefficients were small.

For egg consumption, multivariable linear regression analyses indicated that among urban boys, higher egg intake frequency was positively associated with BMI and FVC. Among urban girls, egg intake was independently associated with FVC and 1-min sit-ups, and was inversely associated with 50 m sprint performance. Among rural boys, egg consumption was positively correlated with BMI and FVC, and inversely associated with 1,000 m running performance. Among rural girls, egg intake frequency was positively associated with FVC, 1-min sit-ups, and BMI, with all associations characterized by small effect sizes.

Regarding dairy product consumption, multivariable linear regression analyses indicated that among urban boys, higher intake frequency was positively associated with FVC and standing long jump performance, and inversely associated with both 50 m sprint and 1,000 m running times. Among urban girls, dairy intake was associated with a broader range of physical fitness, including BMI, FVC, standing long jump, 1-min sit-ups, and both 50 m sprint and 800 m running performance. In the rural group, boys showed positive associations between dairy consumption and FVC, standing long jump, and chin-up performance, along with inverse associations with 50 m sprint and 1,000 m running times. Rural girls exhibited positive associations between dairy intake and FVC, standing long jump, and 1-min sit-ups, and inverse associations with 800 m running time and 50 m sprint time, with all effect sizes remaining small.

Compared with other dietary behaviors, higher SSB intake connected with worse physical fitness outcomes after adjustment for physical activity, sleep duration, and sedentary time. Similar patterns were observed across most urban–rural and sex subgroups. In the urban boys group, higher SSB consumption was inversely correlated with FVC and BMI, and positively correlated with chinups. Among urban girls, SSB intake was inversely associated with FVC, sit-and-reach performance, standing long jump, and 1-min sit-ups, and positively associated with 800 m running time. In the rural group, boys demonstrated inverse associations between SSB intake and FVC, standing long jump, sit and reach, and BMI, while 1,000 m running time and 50 m sprint time were positively associated. Similarly, rural girls exhibited inverse associations between SSB intake and FVC, sit-and-reach performance, standing long jump, and 1-min sit-ups, while 800 m running time was positively associated (Supplementary Table S1).

## 4 Discussion

This study found that, adjusted for physical activity, sleep duration, and sedentary time, selected dietary behaviors, including more frequent breakfast intake, egg consumption, and dairy consumption, were generally associated with more favorable physical fitness across subgroups. In contrast, higher intake of SSBs was associated with poorer fitness outcomes. These associations were more observed across a broader range of outcomes among rural adolescents, particularly girls.

Urban adolescents in our study reported more frequencies of breakfast, egg, and dairy consumption than their rural counterparts, which is consistent with previous reports showing greater egg and dairy intake among urban children (38, 39). Dietary patterns in rural China have changed in recent years, but differences between urban and rural areas remain. These differences are linked to levels of urbanization, income, and local food supply (40). In the past, rural diets relied more on grains. Urban residents usually consumed more dairy products (41). Eggs and dairy products provide protein and calcium, which are linked to muscle strength and physical performance in adolescents (42). This could help to explain why students in urban outperformed those in rural areas on strength tests like sit-ups and chin-ups.

More frequent consumption of breakfast, eggs, and dairy products was associated with better performance on speed, strength, and endurance measures in adolescents. These patterns were observed in both urban and rural groups and among boys and girls. The relationships were stronger in rural adolescents, especially girls. These results are consistent with our original hypothesis and show that these dietary behaviors are related to physical fitness in adolescents (43). Breakfast is an important meal for daily energy intake and normal metabolic function (44). Regular breakfast intake was associated with better performance on several fitness measures, including strength, speed, and endurance, in this study (45). Other studies report similar results. Skipping breakfast is linked to poorer weight control. Regular breakfast intake may help reduce the risk of obesity in children (46). These findings suggest that breakfast intake is related to physical fitness in adolescents and should be studied further in long-term and intervention research. Egg and dairy intake were also related to physical fitness. These foods provide protein, choline, and vitamin D (47). Egg intake contributes essential nutrients during growth and development in adolescents (48). Adequate protein intake provides amino acids required for muscle maintenance and function, which may underlie the observed associations with strength and endurance-related indicators (42). In addition, dairy products also provide calcium and vitamins D and A. These nutrients support bone development and neuromuscular function (49). These results indicate that egg and dairy intake is associated with muscle strength and endurance in adolescents.

SSBs, including soda, flavored juices, energy and sports drinks, and sweetened coffee or tea, are a significant source of added sugar in children’s diets. Frequent intake of SSBs is linked to weight gain and a higher risk of obesity (50), often discussed in relation to excessive energy intake and lower nutrient density (29). About two-thirds of American children consume at least one SSB each day (51). This habit can affect normal metabolic function and overall health (52). SSB intake may also affect health through mechanisms other than calorie intake. High sugar content can lead to high glycemic load and strong insulin responses, which may increase obesity risk (50). In this study, higher SSB intake was linked to poorer physical fitness. Lower performance was observed in measures of muscle strength, endurance, cardiorespiratory fitness, and flexibility (53).

Dietary behaviors were more consistently associated with physical fitness in rural adolescents, particularly in measures of strength and endurance, with these associations being more evident among girls. Although statistically significant, the observed associations were characterized by small effect sizes, suggesting limited practical impact at the individual level. Similar patterns have been reported in recent population studies, which found stronger links between diet and physical fitness in rural children and adolescents (54, 55). Rural adolescents often consume less protein and calcium-rich foods, such as eggs and dairy products (56). Their overall nutritional status may also be lower (56). These factors may make them more sensitive to changes in diet (57). Among girls, muscle strength and endurance can be affected by protein intake to some degree, which may help explain why the associations were stronger in this group.

This study has several limitations. First, data on pubertal maturation stages were not available. Puberty is an important period for adolescents aged 13–16 years. Hormonal changes during this stage may affect both dietary behaviors and physical development. Second, dietary behaviors were measured using only four questionnaire items. These items may not reflect overall diet patterns or detailed nutrient intake. Finally, the study used a cross-sectional design. This design does not allow conclusions about cause and effect. Future studies should include information on pubertal stage, use more detailed dietary measures, and apply longitudinal designs to better examine these relationships.

## 5 Conclusion

Higher consumption of breakfast, eggs, and milk, and reduced intake of SSBs were associated with modestly better physical fitness outcomes among adolescents aged 13–16. The associations were stronger among rural adolescents, particularly among girls. Higher frequencies of breakfast, egg, and dairy consumption were associated with more favorable levels of strength, speed, and endurance, whereas frequent intake of SSBs was associated with poorer physical fitness.

## Data Availability

The datasets presented in this article are not readily available due to confidentiality reasons. Requests to access the datasets should be directed to ysun@snnu.edu.cn.
