# Correction: Dietary behaviors and physical fitness among Chinese adolescents aged 13–16 years: a comparative study on breakfast, eggs, dairy, and sugar-sweetened beverages by urban–rural location and sex

**DOI:** 10.3389/fnut.2026.1798857

**Published:** 2026-02-13

**Authors:** Youjia Li, Shaokai He, Liangsen Wang, Wenyue Ma, Wenfei Zhu, Ying Hou, Yuliang Sun

**Affiliations:** 1 School of Physical Education, Shaanxi Normal University, Xi'an, China; 2 Fuzhou Preschool Education College, Fuzhou, China

**Keywords:** adolescents, Chinese, dietary behaviors, physical fitness, urban–rural

Supplementary Table S1 was erroneously published with the original version of this paper. Some statistically significant results were not clearly indicated, which could lead to misinterpretation of the study findings. The file has now been replaced with a corrected version in which statistically significant values are properly highlighted.

In the published article, there was an error in **Results**, in *Section 3.2* “*Comparison of four dietary behaviors and physical fitness between urban and rural adolescents*.” Some of the results were not described with sufficient clarity. The incorrect paragraph read as follows:

“Significant urban–rural disparities were observed in four dietary behaviors and physical fitness (Table 2). Among boy students, urban residents reported significantly higher frequency of consumption of breakfast (*p* < 0.001, *d* = 0.10), eggs (*p* < 0.001, *d* = 0.162), and dairy products (*p* < 0.001, *d* = 0.238). Additionally, they exhibited higher BMI values (*p* < 0.001, *d* = 0.058) than their rural counterparts. Additionally, urban boys exhibited greater FVC than rural boys (*p* < 0.001, *d* = 0.081). However, urban boys performed significantly poorer than rural boys in both the sit-and-reach test (*p* < 0.001, *d* = −0.065) and the pull-up test (*p* < 0.001, *d* = −0.061). Additionally, urban boys engaged in significantly more time in physical activity than rural boys (*p* < 0.001, *d* = 0.084). Urban girl students demonstrated significantly better performance in FVC (*p* < 0.001, *d* = 0.108) and sit-up tests (*p* < 0.001, *d* = 0.175), along with higher consumption frequencies of breakfast (*p* < 0.001, *d* = 0.109), eggs (*p* < 0.001, *d* = 0.167), and dairy products (*p* < 0.001, *d* = 0.217). Additionally, urban girls exhibited a slightly but statistically higher BMI than their rural counterparts (*p* = 0.046, *d* = 0.026).”

The correct paragraph should read as, “Significant urban–rural disparities were observed in four dietary behaviors and physical fitness (Table 2). Among boy students, urban residents reported significantly higher frequency of consumption of breakfast (*p* < 0.001, *d* = 0.10), eggs (*p* < 0.001, *d* = 0.162), dairy products (*p* < 0.001, *d* = 0.238), and significantly lower sugary beverage consumption (*p* < 0.001, *d* = −0.052). Additionally, they exhibited higher BMI values (*p* < 0.001, *d* = 0.058) than their rural counterparts. Additionally, urban boys exhibited greater FVC than rural boys (*p* < 0.001, *d* = 0.081). However, urban boys performed significantly poorer than rural boys in both the sit-and-reach test (*p* < 0.001, *d* = −0.065) and the chin-up test (*p* < 0.001, *d* = −0.061). Additionally, urban boys engaged in significantly more time in physical activity than rural boys (*p* < 0.001, *d* = 0.084). Urban girl students demonstrated significantly better performance in FVC (*p* < 0.001, *d* = 0.108) and sit-up tests (*p* < 0.001, *d* = 0.175), along with higher consumption frequencies of breakfast (*p* < 0.001, *d* = 0.109), eggs (*p* < 0.001, *d* = 0.167), and dairy products (*p* < 0.001, *d* = 0.217). However, urban girls had longer 800 m running times than rural girls (*p* < 0.001, *d* = 0.079). Additionally, urban girls exhibited a slightly but statistically higher BMI than their rural counterparts (*p* = 0.046, *d* = 0.026).”

In the published article, there was an error in **Results**, *Section 3.3 “Four dietary behaviors and physical fitness in urban versus rural adolescents*,” paragraph 2–5. Certain relationships were not described with sufficient clarity. The incorrect paragraphs read as follows: “In the urban group, breakfast consumption frequency in boys was independently and positively associated with standing long jump performance. Among urban girls, breakfast intake was positively associated with physical fitness, including the sit-and-reach test and the standing long jump. In contrast, inverse associations were observed for running performance. In the rural group, distinct behaviors were observed between boys and girls. Among rural boys, breakfast consumption frequency was positively associated with standing long jump and sit-and-reach performance, while no significant associations were observed with FVC or running-related indicators. In contrast, rural girls showed a broader behavior of associations, with breakfast consumption positively associated with FVC, standing long jump, sit-and-reach performance, and 1-min sit-ups, and negatively associated with both the 50 m sprint and the 800 m run. However, all standardized regression coefficients were small.

For egg consumption, multivariable linear regression analyses indicated that among urban boys, higher egg intake frequency was positively associated with BMI and FVC. Among urban girls, egg intake was independently associated with FVC and 1-min sit-ups, and was inversely associated with 50 m sprint performance. Among rural boys, egg consumption was positively correlated with BMI and FVC, and adversely connected with 1,000 m running performance. Among rural girls, egg intake frequency was positively associated with FVC and 1-min sit-ups, with all associations characterized by small effect sizes.

Regarding dairy product consumption, multivariable linear regression analyses indicated that among urban boys, higher intake frequency was positively associated with FVC and standing long jump performance, and inversely associated with both 50 m sprint and 1,000 m running times. Among urban girls, dairy intake was associated with a broader range of physical fitness, including BMI, FVC, standing long jump, 1-min sit-ups, and both sprint and middle-distance running performance. In the rural group, boys showed positive associations between dairy consumption and FVC, standing long jump, and chin-up performance, along with inverse associations with 50 m sprint and 1,000 m running times. Rural girls exhibited positive associations between dairy intake and FVC, standing long jump, and 1-min sit-ups, and an inverse association with 800 m running time, with all effect sizes remaining small.

Compared with other dietary behaviors, higher SSB intake connected with worse physical fitness outcomes after adjustment for physical activity, sleep duration, and sedentary time. Similar patterns were observed across most urban–rural and sex subgroups. In the urban boys group, higher SSB consumption was inversely correlated with FVC. Among urban girls, SSB intake was inversely associated with FVC, sit-and-reach performance, standing long jump, and 1-min sit-ups, and positively associated with 800 m running time. In the rural group, boys demonstrated inverse associations between SSB intake and FVC, standing long jump, and BMI, while 1,000 m running times were positively associated. Similarly, rural girls exhibited inverse associations between SSB intake and FVC, sit-and-reach performance, standing long jump, and 1-min sit-ups (**Supplementary Table S1**).”

The correct paragraphs should read as, “In the urban group, breakfast consumption frequency in boys was independently and positively associated with standing long jump performance. Among urban girls, breakfast intake was positively associated with physical fitness, including the sit-and-reach test and the standing long jump. In contrast, inverse associations were observed for the 800 m running. In the rural group, distinct behaviors were observed between boys and girls. Among rural boys, breakfast consumption frequency was positively associated with standing long jump and sit-and-reach performance, while no significant associations were observed with FVC or running-related indicators. In contrast, rural girls showed a broader behavior of associations, with breakfast consumption positively associated with FVC, standing long jump, sit-and-reach performance, and 1-min sit-ups, and negatively associated with both the 50 m sprint and the 800 m run. However, all standardized regression coefficients were small.

For egg consumption, multivariable linear regression analyses indicated that among urban boys, higher egg intake frequency was positively associated with BMI and FVC. Among urban girls, egg intake was independently associated with FVC and 1-min sit-ups, and was inversely associated with 50 m sprint performance. Among rural boys, egg consumption was positively correlated with BMI and FVC, and inversely associated with 1,000 m running performance. Among rural girls, egg intake frequency was positively associated with FVC, 1-min sit-ups, and BMI, with all associations characterized by small effect sizes.

Regarding dairy product consumption, multivariable linear regression analyses indicated that among urban boys, higher intake frequency was positively associated with FVC and standing long jump performance, and inversely associated with both 50 m sprint and 1,000 m running times. Among urban girls, dairy intake was associated with a broader range of physical fitness, including BMI, FVC, standing long jump, 1-min sit-ups, and both 50 m sprint and 800 m running performance. In the rural group, boys showed positive associations between dairy consumption and FVC, standing long jump, and chin-up performance, along with inverse associations with 50 m sprint and 1,000 m running times. Rural girls exhibited positive associations between dairy intake and FVC, standing long jump, and 1-min sit-ups, and inverse associations with 800 m running time and 50 m sprint time, with all effect sizes remaining small.

Compared with other dietary behaviors, higher SSB intake connected with worse physical fitness outcomes after adjustment for physical activity, sleep duration, and sedentary time. Similar patterns were observed across most urban–rural and sex subgroups. In the urban boys group, higher SSB consumption was inversely correlated with FVC and BMI, and positively correlated with chin-ups. Among urban girls, SSB intake was inversely associated with FVC, sit-and-reach performance, standing long jump, and 1-min sit-ups, and positively associated with 800 m running time. In the rural group, boys demonstrated inverse associations between SSB intake and FVC, standing long jump, sit and reach, and BMI, while 1,000 m running time and 50 m sprint time were positively associated. Similarly, rural girls exhibited inverse associations between SSB intake and FVC, sit-and-reach performance, standing long jump, and 1-min sit-ups, while 800 m running time was positively associated (**Supplementary Table S1**).”

The original version of this article has been updated.

