# Supplementary material for: Dietary behaviors and physical fitness among Chinese adolescents aged 13–16 years: a comparative study on breakfast, eggs, dairy, and sugar-sweetened beverages by urban–rural location and sex
Source: Front Nutr. 2026 Jan 28;13:1724764. doi: 10.3389/fnut.2026.1724764 (PMC12890674; doi:10.3389/fnut.2026.1724764)
Supplement: Supplementary file 1 [file Table_1.DOCX]

Supplementary Table S1**.** Multivariable linear regression of associations between four dietary behaviors and physical fitness among urban and rural adolescents

|  |  | **Breakfast Consumption** | | | **Egg Intake** | | | **Dairy Product Intake** | | | **Sugar-sweetened beverages Intake** | | |
| --- | --- | --- | --- | --- | --- | --- | --- | --- | --- | --- | --- | --- | --- |
|  | **Outcome** | **β** | ***p*-Value** | **95%CI** | **β** | ***p-*Value** | **95%CI** | **β** | ***p*-Value** | **95%CI** | **β** | ***p*-Value** | **95%CI** |
| **Urban Boys** | **BMI** | 0.006 | 0.521 | (-0.013, 0.025) | 0.024 | **0.016** | (0.005, 0.044) | 0.004 | 0.705 | (-0.016, 0.024) | -0.020 | **0.028** | (-0.038, -0.002) |
|  | **FVC** | -0.014 | 0.142 | (-0.033, 0.005) | 0.084 | **<0.001** | (0.065, 0.103) | 0.026 | **0.008** | (0.007, 0.046) | -0.032 | **<0.001** | (-0.050, -0.014) |
|  | **Sit and reach** | 0.004 | 0.657 | (-0.015, 0.024) | -0.013 | 0.210 | (-0.032, 0.007) | 0.006 | 0.580 | (-0.014, 0.025) | 0.000 | 0.961 | (-0.019, 0.018) |
|  | **Standing long jump** | 0.025 | **0.012** | (0.006, 0.044) | -0.004 | 0.714 | (-0.023, 0.016) | 0.039 | **<0.001** | (0.020, 0.059) | -0.014 | 0.118 | (-0.032, 0.004) |
|  | **50 m sprint** | 0.008 | 0.408 | (-0.011, 0.027) | 0.006 | 0.574 | (-0.014, 0.025) | -0.043 | **<0.001** | (-0.063, -0.023) | -0.006 | 0.491 | (-0.024, 0.012) |
|  | **Chin-ups** | 0.019 | 0.058 | (-0.001, 0.038) | -0.004 | 0.676 | (-0.024, 0.016) | -0.001 | 0.887 | (-0.021, 0.019) | 0.021 | **0.027** | (0.002, 0.039) |
|  | **1000 m running** | -0.015 | 0.117 | (-0.035, 0.004) | -0.016 | 0.102 | (-0.036, 0.003) | -0.044 | **<0.001** | (-0.064, -0.025) | 0.009 | 0.323 | (-0.009, 0.027) |
| **Rural Boys** | **BMI** | -0.003 | 0.746 | (-0.024, 0.017) | 0.057 | **<0.001** | (0.036, 0.077) | -0.010 | 0.356 | (-0.030, 0.011) | -0.035 | **<0.001** | (-0.054, -0.016) |
|  | **FVC** | 0.013 | 0.221 | (-0.008, 0.033) | 0.086 | **<0.001** | (0.065, 0.106) | 0.034 | **0.001** | (0.014, 0.054) | -0.079 | **<0.001** | (-0.097, -0.060) |
|  | **Sit and reach** | 0.034 | **0.001** | (0.013, 0.054) | -0.001 | 0.909 | (-0.022, 0.020) | -0.001 | 0.908 | (-0.022, 0.019) | -0.020 | **0.041** | (-0.039, -0.001) |
|  | **Standing long jump** | 0.021 | **0.037** | (0.001, 0.042) | 0.020 | 0.061 | (-0.001, 0.040) | 0.064 | **<0.001** | (0.044, 0.085) | -0.040 | **<0.001** | (-0.059, -0.021) |
|  | **50 m sprint** | -0.017 | 0.109 | (-0.037, 0.004) | -0.018 | 0.088 | (-0.038, 0.003) | -0.099 | **<0.001** | (-0.120, -0.079) | 0.023 | **0.017** | (0.004, 0.042) |
|  | **Chin-ups** | -0.005 | 0.623 | (-0.026, 0.015) | 0.004 | 0.722 | (-0.017, 0.025) | 0.032 | **0.003** | (0.011, 0.052) | 0.012 | 0.228 | (-0.007, 0.031) |
|  | **1000 m running** | -0.008 | 0.455 | (-0.028, 0.013) | -0.037 | **<0.001** | (-0.058, -0.017) | -0.070 | **<0.001** | (-0.091, -0.050) | 0.026 | **0.007** | (0.007, 0.045) |
| **Urban Girls** | **BMI** | 0.006 | 0.556 | (-0.013, 0.025) | 0.013 | 0.196 | (-0.007, 0.032) | -0.034 | **0.001** | (-0.053, -0.015) | -0.004 | 0.699 | (-0.022, 0.014) |
|  | **FVC** | -0.007 | 0.485 | (-0.026, 0.012) | 0.070 | **<0.001** | (0.051, 0.090) | 0.039 | **<0.001** | (0.019, 0.058) | -0.037 | **<0.001** | (-0.054, -0.019) |
|  | **Sit and reach** | 0.034 | **0.001** | (0.014, 0.053) | 0.002 | 0.811 | (-0.017, 0.022) | 0.019 | 0.055 | (0.000, 0.038) | -0.035 | **<0.001** | (-0.052, -0.017) |
|  | **Standing long jump** | 0.030 | **0.002** | (0.011, 0.049) | -0.009 | 0.352 | (-0.029, 0.010) | 0.055 | **<0.001** | (0.035, 0.074) | -0.047 | **<0.001** | (-0.065, -0.029) |
|  | **50 m sprint** | -0.013 | 0.193 | (-0.032, 0.006) | -0.022 | **0.029** | (-0.041, -0.002) | -0.049 | **<0.001** | (-0.069, -0.030) | 0.007 | 0.477 | (-0.012, 0.025) |
|  | **1 min Sit-ups** | 0.014 | 0.142 | (-0.005, 0.033) | 0.050 | **<0.001** | (0.031, 0.069) | 0.102 | **<0.001** | (0.083, 0.121) | -0.037 | **<0.001** | (-0.055, -0.019) |
|  | **800 m running** | -0.034 | **<0.001** | (-0.053, -0.015) | 0.014 | 0.144 | (-0.005, 0.034) | -0.056 | **<0.001** | (-0.075, -0.037) | 0.044 | **<0.001** | (0.026, 0.062) |
| **Rural Girls** | **BMI** | 0.007 | 0.472 | (-0.012, 0.026) | 0.026 | **0.011** | (0.006, 0.045) | -0.008 | 0.417 | (-0.028, 0.011) | -0.006 | 0.541 | (-0.024, 0.013) |
|  | **FVC** | 0.033 | **0.001** | (0.014, 0.052) | 0.080 | **<0.001** | (0.060, 0.099) | 0.040 | **<0.001** | (0.021, 0.060) | -0.066 | **<0.001** | (-0.084, -0.048) |
|  | **Sit and reach** | 0.041 | **<0.001** | (0.021, 0.060) | 0.010 | 0.312 | (-0.010, 0.030) | 0.017 | 0.088 | (-0.003, 0.036) | -0.041 | **<0.001** | (-0.059, -0.022) |
|  | **Standing long jump** | 0.041 | **<0.001** | (0.022, 0.060) | 0.011 | 0.272 | (-0.009, 0.031) | 0.048 | **<0.001** | (0.028, 0.067) | -0.024 | **0.009** | (-0.042, -0.006) |
|  | **50 m sprint** | -0.044 | **<0.001** | (-0.063, -0.025) | -0.018 | 0.072 | (-0.038, 0.002) | -0.087 | **<0.001** | (-0.106, -0.067) | 0.002 | 0.803 | (-0.016, 0.020) |
|  | **1 min Sit-ups** | 0.047 | **<0.001** | (0.028, 0.066) | 0.052 | **<0.001** | (0.033, 0.071) | 0.126 | **<0.001** | (0.107, 0.145) | -0.052 | **<0.001** | (-0.070, -0.035) |
|  | **800 m running** | -0.063 | **<0.001** | (-0.082, -0.044) | -0.002 | 0.880 | (-0.021, 0.018) | -0.069 | **<0.001** | (-0.088, -0.050) | 0.021 | **0.023** | (0.003, 0.039) |

Note: Dietary behaviors were derived from questionnaire responses regarding the frequency of breakfast, egg, and dairy product consumption in the past 7 days, as well as sugared beverage intake in the past 30 days; β = standardized regression coefficients; BMI, body mass index; FVC, forced vital capacity. Bold values indicate statistical significance (*p* < 0.05).
